# Bioactive Propolis-Silane System as Antifungal Agent in Lignocellulosic-Polymer Composites

**DOI:** 10.3390/ma15103435

**Published:** 2022-05-10

**Authors:** Majka Odalanowska, Grzegorz Cofta, Magdalena Woźniak, Izabela Ratajczak, Tomasz Rydzkowski, Sławomir Borysiak

**Affiliations:** 1 Institute of Chemical Technology and Engineering, Poznan University of Technology, Berdychowo 4, 60965 Poznan, Poland; slawomir.borysiak@put.poznan.pl; 2 Department of Wood Chemical Technology, Faculty of Forestry and Wood Technology, Poznan University of Life Sciences, Wojska Polskiego 28, 60637 Poznan, Poland; grzegorz.cofta@up.poznan.pl; 3 Department of Chemistry, Faculty of Forestry and Wood Technology, Poznan University of Life Sciences, Wojska Polskiego 75, 60625 Poznan, Poland; magdalena.wozniak@up.poznan.pl (M.W.); izabela.ratajczak@up.poznan.pl (I.R.); 4 Department of Mechanical Engineering, Koszalin University of Technology, Raclawicka 15-17, 75620 Koszalin, Poland; tomasz.rydzkowski@tu.koszalin.pl

**Keywords:** polypropylene composites, wood modification, propolis, structure, mechanical properties

## Abstract

Polymer composites with renewable lignocellulosic fillers, despite their many advantages, are susceptible to biodegradation, which is a major limitation in terms of external applications. The work uses an innovative hybrid propolis-silane modifier in order to simultaneously increase the resistance to fungal attack, as well as to ensure good interfacial adhesion of the filler–polymer matrix. Polypropylene composites with 30% pine wood content were obtained by extrusion and pressing. The samples were exposed to the fungi: white-rot fungus *Coriolus versicolor*, brown-rot fungus *Coniophora puteana*, and soft-rot fungus *Chaetomium globosum* for 8 weeks. Additionally, biological tests of samples that had been previously exposed to UV radiation were carried out, which allowed the determination of the influence of both factors on the surface destruction of composite materials. The X-ray diffraction, attenuated total reflectance–Fourier transform infrared spectroscopy, and mycological studies showed a significant effect of the modification of the lignocellulose filler with propolis on increasing the resistance to fungi. Such composites were characterized by no changes in the supermolecular structure and slight changes in the intensity of the bands characteristic of polysaccharides and lignin. In the case of systems containing pine wood that had not been modified with propolis, significant changes in the crystalline structure of polymer composites were noted, indicating the progress of decay processes. Moreover, the modification of the propolis-silane hybrid system wood resulted in the inhibition of photo- and biodegradation of WPC materials, as evidenced only by a slight deterioration in selected strength parameters. The applied innovative modifying system can therefore act as both an effective and ecological UV stabilizer, as well as an antifungal agent.

## 1. Introduction

In recent years, more and more attention has been paid to ecological aspects and environmental protection. These trends have a significant impact on the materials industry, especially plastics. The new directives and regulations require that the products entering the market are made of renewable or recyclable materials as much as possible. Therefore, composites containing fillers of natural origin are very popular [1,2,3]. These include WPCs (wood polymer composites), which consist of a thermoplastic matrix (most often PP, PE, PVC) and a lignocellulose filler. They have many advantages that make them stand out from other composite materials, especially with synthetic fillers. They are characterized by good mechanical properties, stiffness, ease of processing, no need for impregnation, and a relatively low price. Moreover, the use of lignocellulosic filler makes this material partially biodegradable [4,5,6,7,8]. WPCs have enormous application potential. Currently, they are used primarily in construction and the automotive industry [9,10,11,12]. However, due to the characteristics of the components, there is poor adhesion in WPCs between the filler particles and the polymer chains. Weak interactions have a significant impact on obtaining an even dispersion of the filler in the matrix; moreover, they allow the lignocellulosic fibers to move freely and pull them onto the thermoplastic surface under the influence of stresses [13,14,15,16,17]. The presence of lignocellulosic fibers, especially on the surface of the material, makes WPCs very susceptible to the action of microorganisms including fungi. Many works have demonstrated the negative influence of this destructive factor on the structure and properties of WPCs [7,18,19,20,21]. It has been shown that the action of fungi mainly causes the decomposition of the lignocellulosic component, which can be observed indirectly in the weight loss of the sample. Krause et al. (2019) in their work presented the influence of various types of fungi on the structure and properties of WPCs. They showed that the moisture content of the sample plays a key role in the degradation process [22].

Therefore, the modification of lignocellulosic materials used as fillers in WPCs is constantly being developed in order to improve their resistance to microorganisms, mainly fungi. Among the methods used to improve the fungal durability of wood fillers, there is thermal modification or wood impregnation with various protective agents, often synthetic biocides, such as 3-iodo-2-propynyl butylcarbonate, 2-thiazol-4-yl-1H-benzoimidazole, or 4,5-dichloro-2-octyl-isothiazolone [23,24]. However, legal regulations and the degradation of the natural environment lead to bio-friendly methods of wood treatment and modification being preferred. According to literature data, numerous natural substances or individual compounds isolated from them can be applied as potentially protective agents in bio-friendly wood protection, including natural oils, essential oils, plant extracts, chitosan, caffeine, or propolis [25,26,27,28,29]. Propolis is an interesting plant-derived material with broad antifungal activity, applied in wood protection. Wood treated with propolis extracts showed resistance against brown rot (*Coniophora puteana* and *Nelolentinus lepideus*) and white rot fungi (*Trametes versicolor*) [28,30]. In wood protection, propolis extract has also been used as a constituent of mixtures and was mixed with silver nanoparticles, chitosan, caffeine, and silicon compounds [30,31,32,33]. The pine wood treated with propolis extract and silicon compounds showed resistance against *C. puteana*, an increase in bending strength, and a decrease in hygroscopicity compared to untreated wood and wood treated with propolis extract without silicon compounds [30,34,35]. Moreover, chemical analysis, including infrared spectroscopy, nuclear magnetic resonance, and X-ray fluorescence, indicated that constituents of propolis-silane preparations formed permanent bonds with wood components [30]. The results presented in the literature showed that propolis extract and propolis-silane preparations can be applied in ecological wood protection, and also as modifying agents for wood fillers in WPCs, which, to the best of our knowledge, has not been researched yet [34,35].

Another destructive factor that limits the wider use of WPCs is UV radiation. It initiates the photooxidation of the polyolefins, which reduces the molecular weight of the polymer chains. Photodegradation initially takes place on the surface of the composite; however, with time, the radiation reaches the deeper layers of the WPC. This results in a weakening of the interfacial interactions between the matrix and the filler. This leads to a weakening of the stress transfer capacity of the WPC, and the bending strength and modulus of elasticity are deteriorated [36]. The effect of UV radiation also changes the color of the material. This is a real factor as WPCs are very often used in the production of decorative articles in gardening. Thus, it is one of the basic problems that it tries to eliminate by using various UV protectors. The issue of WPC aging has been addressed in many previous works [37,38,39,40,41]. The change in the properties and color of composites depending on the size of the filler particles is presented in the work of Gunjal et al. It was noticed that the most exposed to the color change are composites containing smaller sizes of filler particles, while the greatest deterioration of mechanical properties was observed in samples with larger filler particles [42]. The discoloration and decay of some of the properties of WPCs were also noticed by Kuka et al. In order to eliminate the destructive effect of UV, they proposed a thermal modification of the lignocellulosic filler. They noticed a partial improvement in WPC photodegrading resistance [43]. Composites containing lignocellulosic fillers are used primarily outdoors, where they are exposed to both the effects of weather and fungi. Therefore, when designing a WPC, both these destructive factors should be taken into account. However, there are few literature reports in which their synergistic effect would be discussed [39,44].

This work is a continuation of our previous research on the use of propolis to modify wood in terms of increasing resistance to fungi [45]. However, the work so far has included testing the biological resistance of only wood raw material after treatment with propolis extract. It is worth noting that the resistance to fungal growth of polymer composites with lignocellulosic fillers after modification with the propolis-silanes hybrid system has not been tested so far. An additional message of the research topic undertaken was the determination of the impact of prior exposure to UV radiation of the composite materials on the development of fungi on their surface. Such an assumption reflecting the actual conditions of use of composite products is extremely important when designing materials with increased resistance to aging processes. The aim of this study was to analyze the influence of the applied modifications of the lignocellulosic filler with the bioactive propolis-silane system on the structure and properties of the obtained composite materials. An important task was to check the antifungal activity of the modifiers used in wood fillers, as well as to check the influence of UV radiation and fungi on the structural changes and functional properties of the obtained polymer composites.

## 2. Materials and Methods

### 2.1. Materials

Scots pine sapwood (*Pinus sylvestris* L.) in the form of sawdust (with grain size below 0.5 mm) was used.

The extract of Polish propolis in 70% ethanol was purchased from PROP-MAD (Poznań, Poland), and silicon compounds (tetraethoxysilane and octyltriethoxysilane) were purchased from Sigma Aldrich (Darmstadt, Germany).

The isotactic polypropylene MOPLEN HP456J produced by Basell Orlen Polyolefins (Plock, Poland) with MFR_230°C/2.16kg_, 3.4 g/10 min, was used as the polymeric matrix.

### 2.2. Wood Treatment

Pine wood sawdust was modified with 15% ethanolic propolis extract (EEP) and a mixture (EEP-silanes) consisting of propolis extract, tetraethoxysilane, octyltriethoxysilane, and 70% ethanol in a volume percent ratio of 15:5:5:75. The wood was treated with EEP and a EEP-silanes formulation in the ratio of 1/25 (*w*/*v*). The reaction was carried out at 20 °C for 2 h with simultaneous stirring. Then, wood was filtered and dried in an air flow at room temperature.

### 2.3. Preparation of Composite Materials

**Figure 1:**
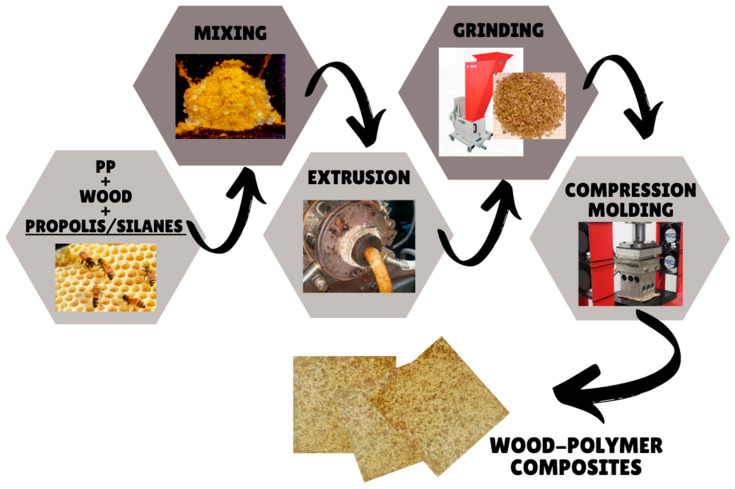
The procedure for the preparation of composites.

The sample preparation procedure was carried out in a laboratory room with a temperature of about 23–24 °C and air humidity at the level of 40–45%. The prepared portions of the composite components (wood and polypropylene) were mixed in a drum mixer until optical homogeneity was obtained. The amount of wood sawdust (modified or not) in the polymer matrix was 30 wt.%. The resulting mixture was then transferred in portions to the hopper of a T-32 screw extruder and extruded. Extrusion parameters: temperature of screw zones: 60; 100; 180; 175 °C. Screw rotation speed: 16 rpm. The composite was extruded through an extrusion head with a circular die with a diameter of 10 mm. The resulting bars were cooled in air. After cooling, the extruded bars were ground in a Wanner 17.26 s high-speed mill to obtain a mill with particles with a diameter ranging from 3 to 7 mm. The shredded composite was then subjected to compression molding, obtaining plates with dimensions of 70 × 70 × 2.5 mm. Compression parameters: temperature, 180 °C; pressure, 500 N/cm^2^. The procedure for composites preparation is illustrated in Figure 1.

### 2.4. UV Aging Tests of Composites Materials

Aging of the composite samples was simulated in an accelerated aging test chamber LU-8047-TM QUV (Q-LAB) spray according to PN-EN ISO 4892-3. The apparatus is equipped with fluorescent lamps emitting UV radiation and a condensation system for the production of dew. The sample degradation process consisted of successive alternating cycles:

1. The dry cycle lasted 8 h, during which the samples were exposed to radiation of the intensity of 0.76 W/m^2^, wavelength of l = 340 nm, and temperature of 60 °C, under the conditions of forced air circulation.
2. The wet cycle lasted 4 h, during which the samples were exposed to steam, at 50 °C, with the fluorescent lamp turned off and under the conditions of forced air circulation.

The radiation dose for the sample irradiated for 24 h was 65.7 kJ/m^2^. Samples for subsequent tests were taken after 7 and 30 days.

### 2.5. Biological Resistance of Composite Materials

The decay resistance of wood plastic composites was performed based on the standard EN 113 with some modifications (sample dimensions and time of exposure to fungi). To investigate the fungal resistance of WPCs, the standard procedure was modified by changing the sample size from 25 by 15 by 50 mm to 4 by 10 by 40 mm. Brown rot fungus (*Coniophora puteana* (Schumacher ex Fries) Karsten (BAM Ebw.15)), white rot fungus (*Coriolus versicolor* (Linnaeus) Quélet (CTB 863 A)), and soft rot fungus (*Chaetomium globosum* Kunze Fries BAM-12) were used to investigate the bio-resistance of composites. Prior to analyses, the composites were dried at the temperature of 70 °C for 18 h and sterilized by a stream of water steam at a temperature of 70 °C for 6 h. Wood composite samples prepared in this way were placed onto the developed mycelium of the test fungus. Next, they were put into a Petri dish and placed in a room ensuring a temperature of 21 °C ± 1 and 75% ± 5 air relative humidity. The mycological test lasted 8 weeks. After the termination of the test, samples were carefully cleaned and mycelium removed and they were dried at 70 °C until reaching constant weight. Composite weight losses were calculated from the weight differences of samples before and after the test.

### 2.6. Characterization of Polymer Composites

#### 2.6.1. Attenuated Total Reflectance–Fourier Transform Infrared Spectroscopy (ATR–FTIR)

The spectra of composites were recorded by a Nicolet iS5 spectrophotometer (Thermo Fisher Scientific, Waltham, MA, USA) with Fourier transform and equipped with a deuterated triglycine sulfate (DTGS) detector and attenuated total reflection (ATR) attachment. The spectra were recorded. Five measurements for each samples, by re-sampling at different locations across entire samples, were recorded over the range of 4000–400 cm^−1^, at a resolution of 4 cm^−1^ and 16 co-added scans. All spectra were given in transmittance units and no ATR baseline correction was applied.

#### 2.6.2. X-ray Powder Diffraction (XRD)

The supermolecular structure of the modified and unmodified composite systems exposed to various destructive factors was analyzed by wide-angle X-ray scattering. Measurements were made at a wavelength of the Cu Kα radiation source of 1.5418 Å at 30 kV and an anodic excitation of 25 mA. The diffractograms of all samples were recorded in the 2θ angle range from 5 to 30° with a step of 0.04°/3 s. The deconvolution of the peaks was performed by the Hindeleh and Johnson method [46,47]. The degree of crystallinity (Xc) of the WPC samples was then determined by comparing the area under the crystalline peaks and the amorphous curve.

#### 2.6.3. Mechanical Tests

The mechanical tests of reference samples and composites after an aging test and after biological tests were carried out in accordance with the PN-EN ISO 527 standard. The endurance tests were performed on the Zwick Z020 universal mechanical testing machine (Zwick/Roell, Ulm, Germany) with a load cell capacity of 20 kN at a cross-head of 5 mm/min. The obtained stress–strain curves were used to determine selected strength parameters such as: Young’s modulus (YM), tensile strength (TS), and elongation at break (EB).

## 3. Results and Discussion

### 3.1. Weight Loss of Composites Caused by Wood-Decaying Fungi

**Figure 2:**
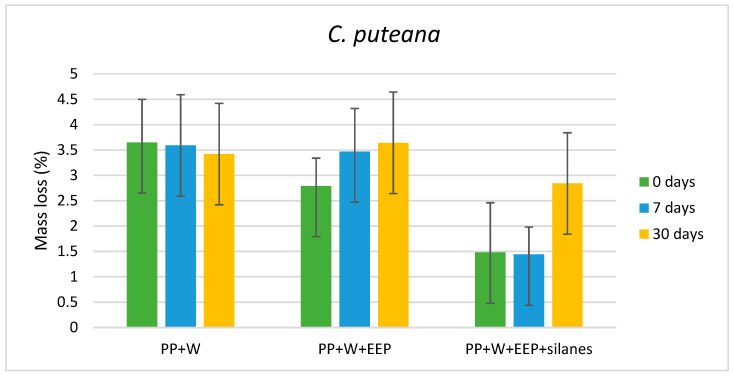
Mass loss of composites after exposure to *C. puteana*.

**Figure 3:**
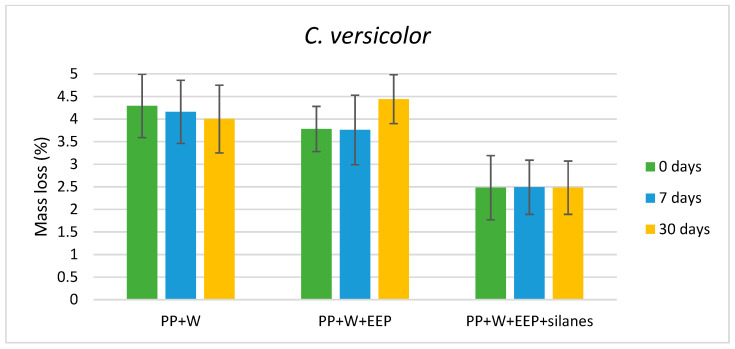
Mass loss of composites after exposure to *C. versicolor*.

The antifungal efficacy of WPCs against wood-destroying fungi, expressed as the average mass loss of composite samples, are presented in Figure 1, Figure 2 and Figure 3. The results showed that composites exhibited the highest resistance against soft rot fungus—*Ch. globosum*, while the durability of WPCs against *C. puteana* and *C. versicolor* was lower and similar for all variants of composites. Moreover, the mycological test indicated that composites containing wood treated with solution consisting of the propolis extract and silanes showed higher resistance against all tested fungi species, compared with composites containing untreated wood or wood treated with propolis extract without silicon compounds.

The weight loss of the composite with untreated wood and wood treated with propolis extract was about 3.5% for both samples before and after the UV aging test and exposure to *C. puteana*, with the exception of the composite containing wood treated with propolis extract and not exposed to UV radiation, for which the weight loss was 2.8% (Figure 2). In turn, the mass loss of the composite containing wood treated with propolis extract and silanes was similar for samples before the aging test and after 7 days of UV radiation and was about 1.5%, whereas the elongation of UV radiation of samples to 30 days caused lower durability against the destructive action of *C. puteana.*

The composite containing wood treated with EEP and silanes showed lower values of mass loss (below 2.5%) and a higher resistance against *C. versicolor* than composites containing untreated wood and wood treated with propolis extract (weight loss about 4%), as presented in Figure 3. The UV aging test did not influence the WPC mass loss values, expected of the composite sample containing wood treated with EEP and subjected to 30 days of UV radiation, where the mass loss of samples was lower than for wood before and after 7 days of aging.

**Figure 4:**
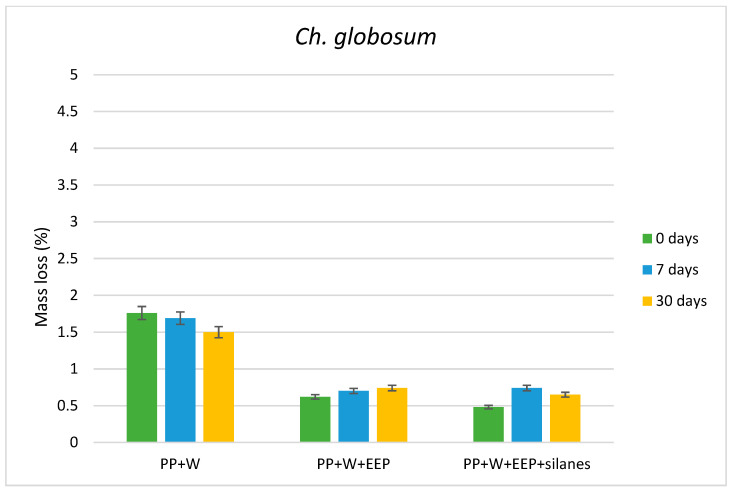
Mass loss of composites after exposure to *Ch. globosum*.

In the case of composites’ exposure to *Ch. globosum*, samples containing wood treated with propolis extract and EEP with silane compounds exhibited a higher resistance against the tested fungus than composites with untreated wood (Figure 4). Moreover, the UV radiation did not influence the mass loss values of composite samples.

In order to increase the resistance of wood to biotic conditions, various types of preservatives are used, and currently, preparations based on natural substances are becoming more and more popular, e.g., propolis extract [27,28]. The literature data confirmed that wood treated with propolis extract and a mixture of propolis extract and silicon compounds showed resistance to wood-decay fungi, including *C. puteana* [28,30,48]. In addition, wood used as a filler in wood composites are often modified or treated with preservatives to obtain resistance of WPCs against wood destructive microorganisms. WPCs with thermally modified wood fibers exhibited an improved resistance against *C. puteana* compared to WPCs with unmodified fibers [49]. Composites consisting of polypropylene with poplar sawdust treated with nano-clay were characterized by a durability against both white rot (*Physisporinus vitreus*, *Pleurotus ostreatus*, and *C.* *versicolor*) and brown rot fungi (*C. puteana* and *Antrodia vaillantii*) compared to WPCs with untreated wood [20]. In turn, Müller et al. [50] investigated the resistance of composites consisting of wood modified with acetic anhydride and treated with methylated melamine-form aldehyde resin or aminosilanes mixed with polyvinyl chloride against basidiomycetes (*C. puteana* and *C. versicolor*), and they found that only aminosilanes-treated WPCs showed a slight decrease in mass loss compared to untreated reference samples of WPCs.

**Figure 5:**
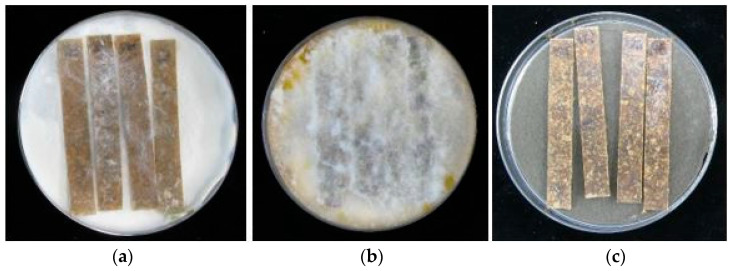
Composite samples (PP+W+EEP+silanes) after UV aging (30 days) and exposure to (**a**) *C. puteana*, (**b**) *C. versicolor*, and (**c**) *Ch. globosum*.

An important factor when choosing a wood treatment preparation that is used as fillers for WPCs seems to be its resistance to abiotic factors, including UV radiation. The results presented in Figure 1, Figure 2 and Figure 3 indicated that WPCs consisting of EEP-treated wood showed lower resistance against *C. puteana* and *C. versicolor*, when the composite samples were exposed to UV radiation for 30 days. In addition, WPCs containing wood treated with EEP and silanes showed an increase in the value of weight loss after UV irradiation for 30 days and exposure to *C. puteana* compared to WPC samples before and after 7 days of UV radiation. However, WPCs containing wood treated with EEP and silanes, also after exposure to UV radiation, showed a high resistance against the tested fungi (Figure 5). The higher resistance of wood treated with the mixture of propolis extract and silicon compounds against UV radiation compared to wood treated with propolis extract without silanes were confirmed in the literature [51].

The highest resistance of WPCs was observed in the case when the samples were exposed to *Ch. globosum*, even when the samples were subjected to UV radiation, which is connected with the action mechanism of this fungus. *Ch. globosum* is a representative of the S1-type soft rot [52], which causes slight losses in the mass of wood, especially coniferous wood, compared to other types of brown rot and white rot fungi. However, with relatively small losses of wood mass, a relatively large loss of physical and mechanical parameters of degraded wood is noticeable, but not as significant as is observed for wood infested with brown rot fungi [53]. For this reason, in the conducted experiment, a significant difference in weight loss was observed between *Ch. globosum* and the other test fungi (*C. puteana* and *C. versicolor*).

### 3.2. Changes in Composite Structure Caused by Decay Fungi

**Figure 6:**
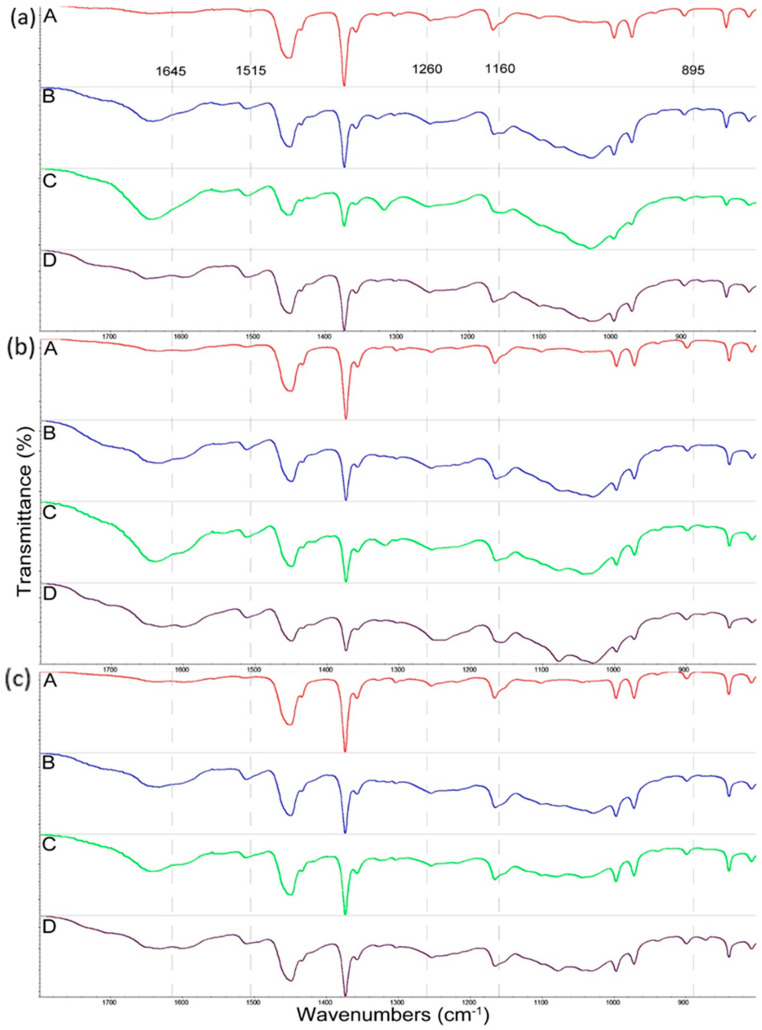
FTIR–ATR spectra of composite samples: (**a**) PP+W (A–), (**b**) PP+W+EEP (A–), and (**c**) PP+W+EEP+silanes (A–), after UV aging (30 days) and exposure to *C. puteana* (B–), *C. versicolor* (C–), and *Ch. globosum* (D–).

The impact of fungal activity in the wood structure was also determined using infrared spectroscopy measurements. In this section, the spectra (Figure 6) of composites after 30 days of UV radiation and exposure to fungi are discussed. The changes in IR spectra of composites before and after 7 days of UV radiation were similar and are presented in Supplementary Materials. Moreover, no change was observed in the spectra of the polypropylene without the wood filler; therefore, the PP spectra are not shown.

The exposure of wood composites to *C. puteana* caused significant changes in the ATR–FTIR spectra of WPC samples, causing changes in the intensities of bands characteristic for polysaccharides and lignin. The bands characteristic for carbohydrates were observed in the IR spectra at 1375 cm^−1^ (deformation of C-H cellulose and hemicelluloses), 1160 cm^−1^ (C-O-C vibration in cellulose and hemicelluloses), and 895 cm^−1^ (C-O-C stretching at β-1,4-glucoside linkages of cellulose and hemicelluloses) [33,54,55,56,57]. In turn, the lignin characteristic bands were presented at 1645 cm^−1^ (conjugated C=O stretching Ph-(C=O)-groups), 1515 cm^−1^ (aromatic skeletal vibration), and 1260 cm^−1^ (guiacyl ring breathing and C=O stretching from lignin) [56,58,59,60]. In the spectra of composites after exposure to *C. puteana* (Figure 6a–c, spectra B), the intensities of carbohydrate bands (1375 cm^−1^, 1160 cm^−1^, and 895 cm^−1^) were lower compared to intensities of these bands in the spectra of composites before exposure to fungus (Figure 6a, spectra A). In contrast, the intensities of lignin bands (1645 cm^−1^, 1515 cm^−1^, and 1260 cm^−1^) slightly increased in the spectra of composites subjected to the activity of *C. puteana*, compared to spectra of composites before fungus action.

Slight differences in the intensities of lignin bands observed in the spectra of composites can be connected with a small amount of wood filler in the composites, and, therefore, a small amount of lignin content in WPCs, possibilities of overlapping bands of wood components, compounds from propolis extract, and a low impact of destructive action of fungus into the wood structure, which confirmed the low weight loss values (Figure 2).

The spectra of composites after exposure to *C. versicolor* presented in Figure 6a–c (spectra C) showed changes in the intensities of structural wood components in comparison to spectra of undecayed WPCs. The intensity of bands resulting from polysaccharides (1375 cm^−1^, 1160 cm^−1^, and 895 cm^−1^) decreased in comparison to the bands in the spectra of undecayed composites. In turn, the intensity of lignin bands at 1515 cm^−1^ in the spectra of composites after exposure to *C. versicolor* was similar to that in the spectra of composites before fungus action, whereas the intensity of bands at 1645 cm^−1^ increased in the spectra of decayed WPCs. The literature data showed that the increase in the 1645 cm^−1^ bands intensities indicate the presence of mycelium formed by *C. versicolor* in decayed wood samples [61]. The changes in decayed composite samples confirmed the ability of *C. versicolor* to utilize all major chemical components in the cell wood wall, suggesting that *C. versicolor* is a nonselective white-rot fungus, especially in the early step of its action, which agrees with literature data [62,63,64,65,66,67].

The exposure of WPC samples to the action of *Ch. globosum* caused a decrease in the intensities of bands corresponding to cellulose and hemicelluloses (1375 cm^−1^, 1160 cm^−1^, and 895 cm^−1^), compared to intensities of these bands in the spectra of undecayed samples, as presented in Figure 6a–c (spectra C). The intensities of the lignin band (1515 cm^−1^) was similar in the spectra of decayed and undecayed composites samples. The ATR-IR results confirmed that *Ch. globosum* had the ability to degrade polysaccharides and lignin, with preference for the former, which agrees with the results presented in the literature [68,69].

The relative intensities of polysaccharides-associated bands at 1375 cm^−1^, 1160 cm^−1^, and 895 cm^−1^ against the lignin band at 1515 cm^−1^ were calculated using peak heights and are presented in Table 1. It can be seen that wood decay caused by all the tested fungi (*C. puteana*, *C. versicolor*, and *Ch. globosum*) resulted in a decrease in carbohydrate/lignin ratio, which indicates that the tested fungi are capable of altering the main components of the cell walls (lignin, cellulose, and hemicelluloses), causing simultaneous wood destruction.

### 3.3. Supermolecular Structure of Polymer Composites after UV Aging and Biological Action

**Figure 7:**
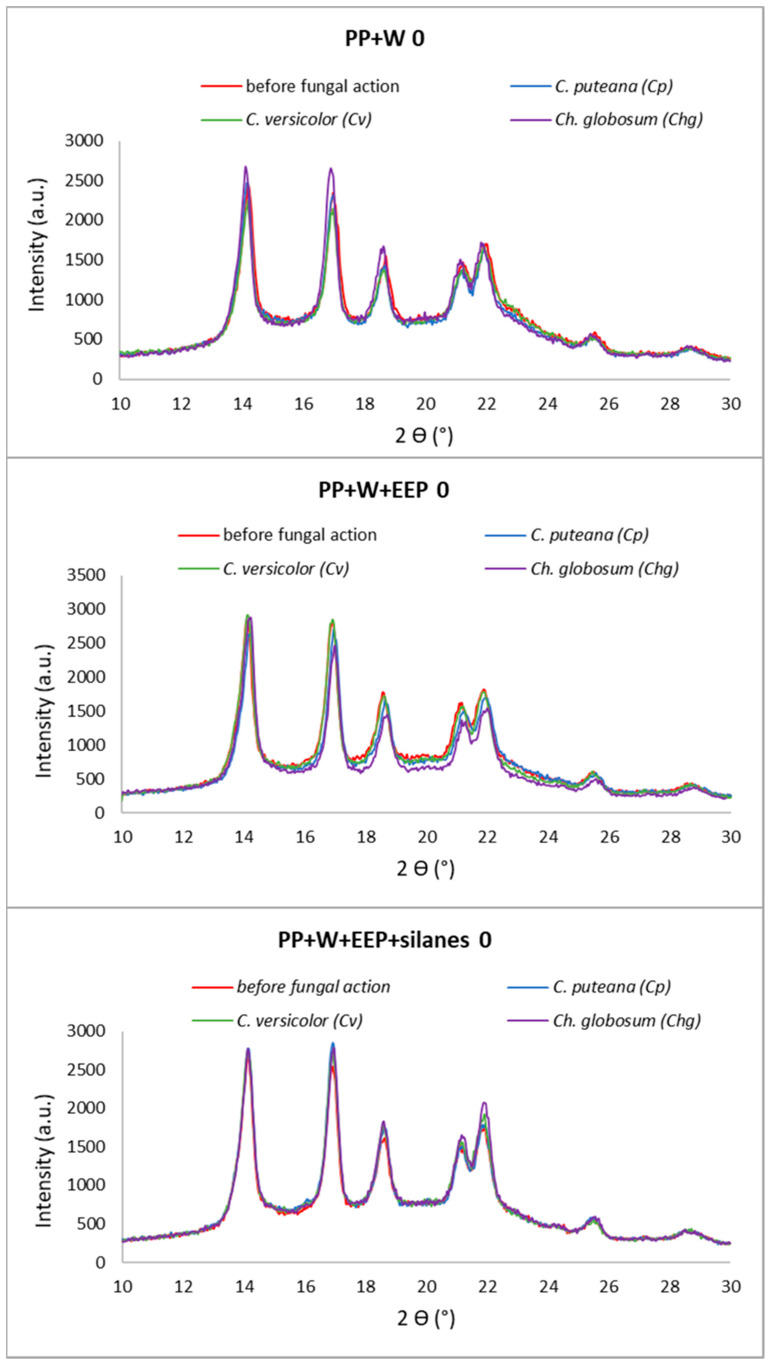
X-ray diffraction pattern of composites without UV aging.

**Figure 8:**
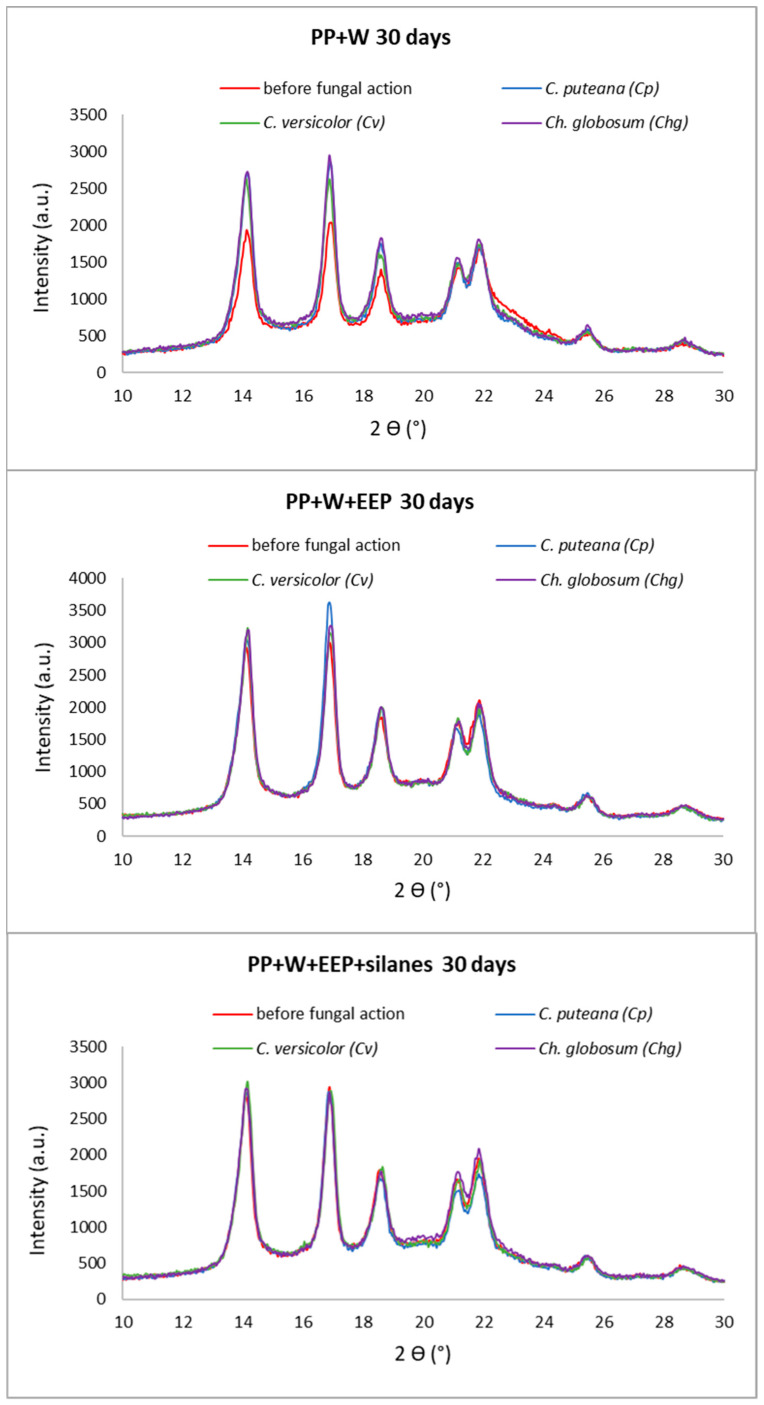
X-ray diffraction pattern of composites after 30 days of UV aging.

The supermolecular structure of PP wood composites was investigated using wide-angle X-ray diffraction (XRD). The diffraction curves obtained for composite systems without and after 30 days of UV irradiation and fungal exposure are shown in Figure 7 and Figure 8.

All the obtained diffractograms show the maxima derived from the α polymorph of polypropylene. This is indicated by the maxima appearing at the diffraction angles 2ϴ = 14°, 17°, 18.5°, 21°, and 22° [70]. Importantly, there were no maxima derived from the β-PP variety. Thus, the introduction of the filler, processing, and action of UV and fungi did not affect the formation of this type of PP.

During structural studies, the influence of two destructive factors (UV and fungi) on the supermolecular structure of composites was analyzed. The analysis of the obtained diffraction curves did not show any significant changes in the supermolecular structure of composites exposed to fungi, without UV. The curves had a similar course. In the case of samples additionally exposed to UV radiation, changes in the supermolecular structure of composites can be noticed. Differences in the intensity of the peaks were noticed depending on the type of fungus. The greatest changes are visible in the case of composites containing raw wood. Based on the analysis of the obtained curves, it can be concluded that the combination of fungal action and UV influences the supermolecular structure of composites. However, in the case of composite samples containing propolis-modified wood, these changes were not significant. In order to explain the obtained relationships in detail, the content of the crystalline phase (Xc) in the composites was determined in the next step. The degree of crystallinity of composite materials is the total result of the content of this phase in the material, both from PP and cellulose. The results are summarized in Table 2.

It can be seen that the modification of wood with propolis and silanes (without UV-radiation and fungi action) resulted in an increase in the Xc value compared to composites containing raw wood. In this case, the increase in the degree of crystallinity caused by the modification of the filler can be explained by the changes taking place during the reaction of wood with the ethanolic propolis solution. During the treatment, low-molecular-weight compounds contained in the wood are washed out and amorphous parts of cellulose are also cracked. These actions increase the proportion of the crystalline phase in the filler. The influence of propolis modification on the supermolecular structure of wood was investigated and explained in the previous work [45].

Exposure of the composites to UV irradiation caused a slight increase in the content of the crystalline phase in relation to the reference samples. However, the introduction of the second factor—fungi—resulted in a large variation in the Xc values. The greatest changes were recorded for samples with raw wood—the degree of crystallinity increased from 32 to even 43%. Interestingly, the action of both factors in the case of composites with wood modified with propolis extract did not cause significant changes in Xc. All PP+W+EEP 30 samples that were bioassayed had comparable Xc. In the case of composites with propolis-modified wood and silanes, the results were practically identical. It can therefore be concluded that in the case of composites containing unmodified wood exposed to both UV and fungi, a synergistic destructive effect of these factors is observed. However, this action is inhibited by the chemical modification of wood with propolis and silanes. Increasing the content of the crystalline phase in samples exposed to UV radiation is related to photooxidation. UV radiation first breaks the bonds in the amorphous part of the matrix. This action ‘exposes’ the wood particles in the composite, which increases their susceptibility to UV radiation. Given that about 2/3 of all wood polysaccharides are amorphous and readily photodegradable, the overall content of the crystalline phase has to increase after irradiation [60,69,71]. The content of the crystalline phase in the sample therefore initially increases and decreases when a certain maximum is reached. The described dependencies were also noticed by Guadagno et al. In their work, they investigated changes in the structure of PP exposed to UV at different times. They noticed a significant increase in the value of Xc (even by 135%) compared to unexposed PP. They explained such large differences with the phenomenon of breaking the polymer chains caused by photooxidation. As a result, the amorphous parts of the chains were shortened, making the remaining parts more mobile and free to crystallize [72]. Similar relationships for PP were also noted by Morancho et al. [73]. On the other hand, the increase in the crystalline cellulose fraction observed in the tested wood sample exposed to UV rays is consistent with that noted by other researchers [74,75]. The stabilization of Xc results for systems containing modified wood can be explained by the protective effect of propolis. The activity of propolis against decay fungi has been studied and published many times [28,30,33]. There have also been many studies showing that propolis is a material capable of absorbing UV radiation [76,77,78]. Propolis works great as a radical scavenger. This work, however, concerned its medical applications. Thus far, it has not been used as a UV protector in composite materials.

Summarizing, the results of XRD studies showed the influence of UV irradiation and fungi on the supermolecular structure of composites. The greatest changes in Xc were observed for samples with unmodified wood treated with UV. However, in the case of introducing propolis into the wood structure, no significant changes in the value of Xc were noticed. The obtained dependencies allow for assumptions that propolis can be used in WPCs both in the form of a UV protector and an antifungal agent.

### 3.4. Mechanical Testing of Polymer Composites after UV Aging and Biological Action

**Figure 9:**
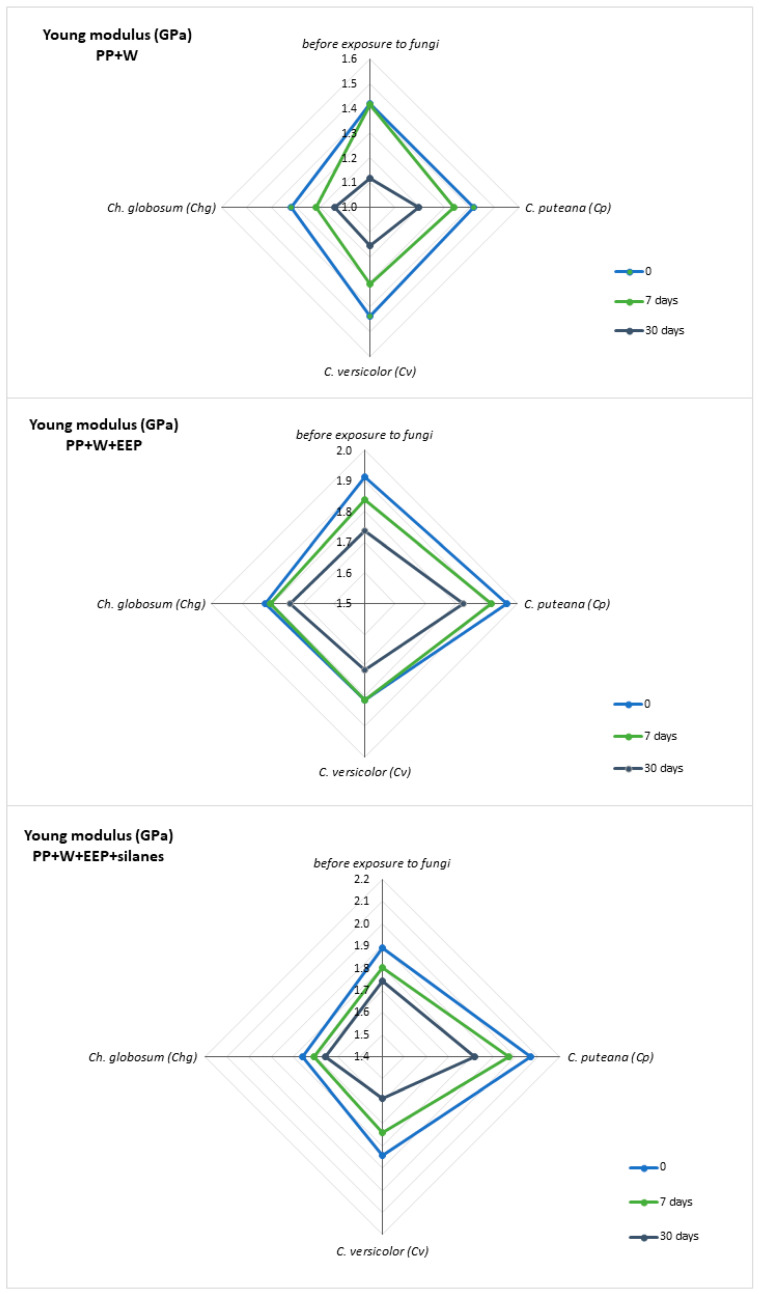
Young’s modulus of composites before and after UV radiation and fungal action.

**Figure 10:**
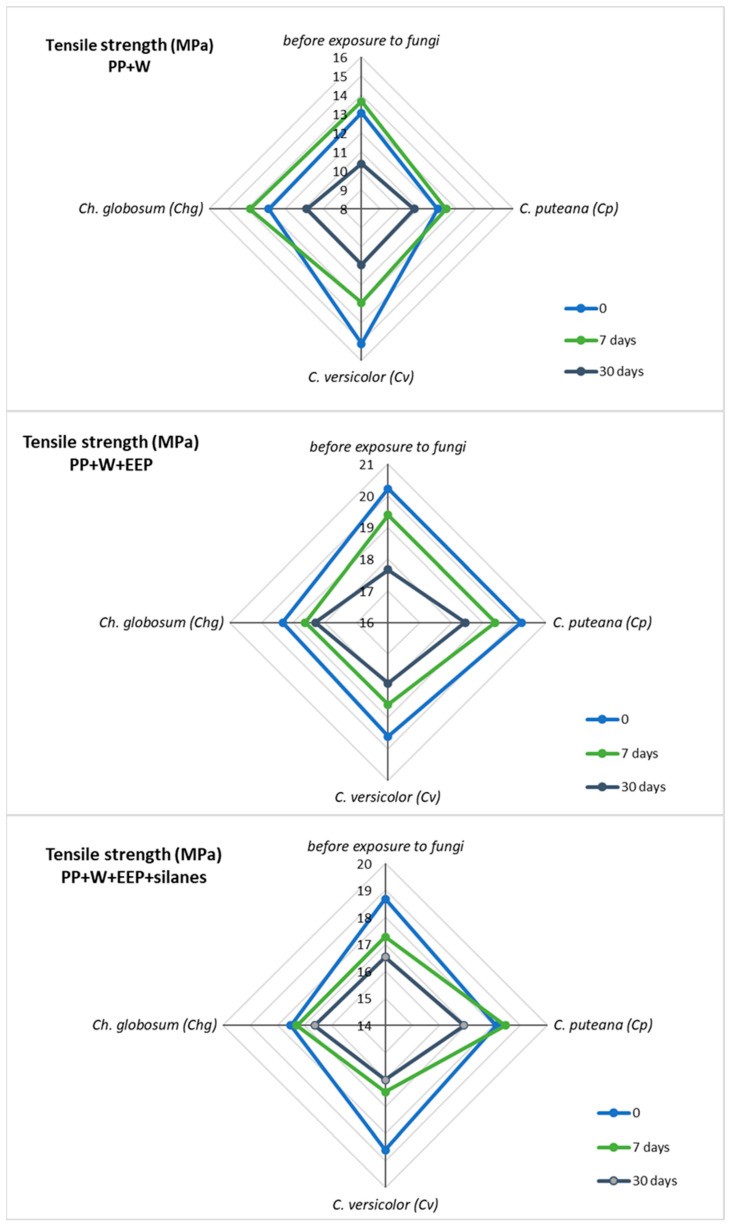
Tensile strength of composites before and after UV radiation and fungal action.

Mechanical tests were carried out to evaluate the effect of UV irradiation and fungal action on the strength properties of the obtained composites. The results for tensile strength, Young’s modulus, and elongation at break are summarized in Table 3, Table 4 and Table 5 and in Figure 9 and Figure 10.

It can be seen that the modification of wood with propolis and silanes improved the mechanical properties of WPCs. The highest values of parameters were obtained for composites with wood modified with propolis, which correlates very well with the XRD results. The highest Xc values were also obtained for samples of this composite. The value of Young’s modulus for the reference sample was 1.42 GPa, while for composites with modified wood, it was about 1.9 GPa.

Mechanical tests of samples exposed to UV treatment confirmed the negative influence of this factor on the strength properties of WPCs. It was noticed that the strength parameters of composites decrease with the time of exposure. The greatest changes in properties were observed for WPC samples with unmodified wood. Young’s modulus and tensile strength values for these composites decreased by over 20%. Wood modification with propolis and silanes resulted in a greater stability of the strength parameters. For these samples, a decrease in YM and TS by only about 10% was observed.

The addition of another destructive factor also resulted in a reduction in the mechanical properties of the samples; however, the effect differed depending on the type of attacking fungus. The WPC samples showed the highest susceptibility to the action of *C. versicolor*, while the least aggressive fungus turned out to be *Ch. globosum.* However, the destructive effect of fungi on the strength parameters was limited in the composite samples containing propolis-treated wood. Interestingly, both UV radiation and the action of all types of fungi did not have a significant effect on the elongation of break of all tested composites.

The obtained relationships are perfectly illustrated in Figure 9 and Figure 10, where the change in the values of individual parameters under the influence of two parameters—UV radiation and fungal activity—is shown. It can be seen that there is a large discrepancy in the values for PP+W composites. These composites are very susceptible to both fungi and UV radiation, and the action of both of these factors increases their destructive effect and the reduction in the YM and TS values. However, for the remaining composites containing propolis, an inhibitory effect was noticed. This proves the beneficial effect of propolis and the increased protection of the material against both factors. The decrease in the strength of composites can be explained by the photodegradation of the material.

The obtained dependencies are consistent with the literature data. Many studies have shown that UV radiation played a remarkable role in the decline in the mechanical properties of composite materials. In these works, it was found that the tensile strength of the fibers decreases with exposure time [10,79,80]. An extensive review of the effects of weathering on the properties of WPCs was presented by Friedrich [36]. The effect of coupling agents (maleic anhydride-grafted polypropylene and m-TMI grafted polypropylene) on the mechanical properties of WPCs after UV was investigated by Gunjal et al. [42]. The influence of fungi on WF/HDPE composites in the presence of various compounds was also investigated by Feng et al. [19]. They noticed a decrease in the strength parameters of composites exposed to mycelium. The properties, however, improved when fungicides were added to the system. Curling et al. presented the relationship between the mechanical properties, weight loss, and chemical composition of wood caused by brown rot decay. In the work, they investigated the influence of degradation on the composition of hemicellulose and the relationship between the decay and the mechanical properties of wood [81]. The results obtained in our study indicate a positive effect of propolis on the protection of wood against fungi. This phenomenon was demonstrated in many previous works, but they concerned only solid wood. Akcay et al. in their research used propolis methanol extract (MPE) as an antifungal agent for the preservation of Scots pine wood. They observed a 91.5% degree of protection against wood rot fungus compared to the control [28]. Woźniak et al. and our previous work also confirmed the effectiveness of improving the resistance of wood to fungi (including *C. puteana*) as a result of the action of propolis [45,48].

Summing up, the analysis of the obtained results of mechanical tests showed that the greatest deterioration of the strength properties was noted for raw wood systems exposed to both fungi and UV. The modification of wood with propolis extract and silanes resulted in the production of composites with a much greater resistance to the action of destructive factors, as evidenced only by a slight deterioration in selected strength parameters.

## 4. Conclusions

The conducted research showed a significant effect of the wood modification with propolis extract on increasing the fungal resistance of the obtained composite materials. The composites containing wood impregnated with the propolis-silanes formulation showed the highest resistance against all tested fungi (*Ch. globosum*, *C. puteana*, and *C. versicolor*), also when composite samples were subjected to UV radiation. The higher resistance of these WPCs may be connected with the synergistic action of the bioactive components of propolis and silicon compounds, which possesses hydrophobic properties, and thus may reduce the moisture content of wood. The UV radiation did not influence the WPC resistance against *T. versicolor* and *Ch. globosum*. Only in the case of the WPC with wood treated with the propolis-silanes system and exposed to *C. puteana*, a slight increase in weight loss of WPC samples after 30 days of radiation was observed. The resistance of tested composites, especially in the case of composites containing wood impregnated with propolis extract and silicon compounds, against decay fungi was also confirmed by ATR–FTIR analysis, where slight changes in the intensities of bands characteristic for polysaccharides and lignin were observed. Moreover, the relative intensities of polysaccharides bands against the lignin band indicated that the action of tested fungi resulted in a decrease in carbohydrate/lignin ratio in WPC samples, which confirm that all fungi are capable of removing all chemical components of the cell walls, causing simultaneous wood destruction.

In the further part of the work, structural and mechanical tests of composites containing modified lignocellulosic fillers, previously exposed to UV radiation and fungi, were carried out. Then, the impact of the modification of the filler on the protection of WPCs against the effects of the above-mentioned destructive factors was assessed. The analysis of the XRD test results showed that all types of fungi and UV radiation cause a change in the WPC supermolecular structure, as evidenced by an increase in the Xc value. The range of changes increased with the exposure time and was most noticeable for composites with unmodified wood. However, composite samples containing wood treated with propolis extract and silanes were characterized by Xc values similar to the reference samples. Mechanical tests also showed a synergistic destructive effect of UV radiation and fungi activity on composite materials. For WPC systems with raw wood, a significant deterioration in strength properties was noted. Moreover, it has been shown that the use of propolis extract and the hybrid system (propolis-silanes) in the modification of the filler results in obtaining a material with only a slight deterioration in selected strength parameters (YM, Eb, TS) and a stabilization of the values of these parameters during irradiation.

On the basis of the obtained results, it can be concluded that the introduction of fillers modified with propolis and silanes into the polymer matrix results in the inhibition of photo- and biodegradation of WPC materials. Thus, the proposed modifying system plays the role of an effective and ecological UV stabilizer and an antifungal agent.

## Figures and Tables

**Table 1:** Relative changes in the ratios of carbohydrate bands with lignin reference band in composite samples decayed by wood rot fungi.

Tested Fungus	Composite Samples	I_1375/1515_	I_1160/1515_	I_895/1515_
WPC before fungus exposition	PP+W	20.706	5.202	2.577
	PP+W+EEP	15.716	5.344	0.687
	PP+W+EEP+silanes	12.091	3.549	1.320
*C. puteana*	PP+W	14.646	4.192	1.774
	PP+W+EEP	4.369	2.243	0.430
	PP+W+EEP+silanes	9.770	3.005	1.179
*C. versicolor*	PP+W	8.472	2.743	1.090
	PP+W+EEP	3.887	1.813	0.481
	PP+W+EEP+silanes	4.601	2.021	0.549
*Ch. globosum*	PP+W	7.728	2.997	1.246
	PP+W+EEP	2.213	1.309	0.411
	PP+W+EEP+silanes	4.536	2.101	0.748

**Table 2:** The results of Xc-obtained composites samples.

Composite Samples	Xc (%)			
	Tested Fungus			
	-	*C. puteana*	*C. verisolor*	*Ch. globosum*
PP+W “0”	30	31	28	34
PP+W+EEP “0”	37	35	37	40
PP+W+EEP+silanes “0”	36	38	37	39
PP+W 30 days	32	41	40	43
PP+W+EEP 30 days	40	44	43	43
PP+W+EEP+silanes 30 days	39	38	40	40

**Table 3:** Young’s modulus of WPCs.

Young’s Modulus (GPa)												
	Before Exposure to Fungi			*C. puteana (Cp)*			*C. versicolor (Cv)*			*Ch. globosum (Chg)*		
Time of UV Irradiation (Days)	0	7	30	0	7	30	0	7	30	0	7	30
PP+W	1.42	1.42	1.12	1.42	1.34	1.20	1.44	1.31	1.15	1.32	1.22	1.15
PP+W+EEP	1.91	1.84	1.74	1.96	1.91	1.82	1.82	1.82	1.72	1.82	1.81	1.75
PP+W+EEP+silanes	1.89	1.80	1.74	2.07	1.97	1.82	1.84	1.74	1.59	1.76	1.71	1.66

**Table 4:** Tensile strength of WPCs.

Tensile Strength (MPa)												
	Before Exposure to Fungi			*C. puteana (Cp)*			*C. versicolor (Cv)*			*Ch. globosum (Chg)*		
Time of UV Irradiation (Days)	0	7	30	0	7	30	0	7	30	0	7	30
PP+W	13.05	13.66	10.38	12.02	12.46	10.79	15.11	12.95	10.95	12.88	13.86	10.87
PP+W+EEP	20.23	19.39	17.67	20.22	19.39	18.44	19.60	18.59	17.92	19.32	18.61	18.29
PP+W+EEP+silanes	18.68	17.27	16.53	18.11	18.45	16.92	18.61	16.47	16.02	17.50	17.27	16.61

**Table 5:** Elongation at break of WPCs.

Elongation at Break (%)												
	Before Exposure to Fungi			*C. puteana (Cp)*			*C. versicolor (Cv)*			*Ch. globosum (Chg)*		
Time of UV Irradiation (Days)	0	7	30	0	7	30	0	7	30	0	7	30
PP+W	1.65	1.87	1.77	1.56	1.58	1.61	2.07	2.01	1.91	1.78	1.76	1.90
PP+W+EEP	2.21	2.35	1.84	1.83	1.77	1.81	2.11	2.04	1.93	1.84	1.76	1.84
PP+W+EEP+silanes	2.10	2.22	1.58	1.74	1.95	1.71	2.31	2.17	2.27	2.03	2.01	2.04

## Data Availability

The data reported in this study can be available by request from the authors.

## Supplementary Materials

The following supporting information can be downloaded at: https://www.mdpi.com/article/10.3390/ma15103435/s1, Figure S1. FTIR–ATR spectra of composite samples: (a) PP+W (A–), (b) PP+W+EEP (A–), and (c) PP+W+EEP+silanes (A–), without UV aging (0) and exposure to *C. puteana* (B–), *C. versicolor* (C–), and *Ch. Globosum* (D–). Figure S2. FTIR–ATR spectra of composite samples: (a) PP+W (A–), (b) PP+W+EEP (A–), and (c) PP+W+EEP+silanes (A–), after UV aging (7 days) and exposure to *C. puteana* (B–), *C. versicolor* (C–), and *Ch. Globosum* (D–).

## References

[1] Thomas S., Visakh P.M., Mathew A.P. (2012). Advances in Natural Polymers: Composites and Nanocomposites.

[2] Sandberg D., Kutnar A., Mantanis G. (2017). Wood Modification Technologies—A Review. iForest Biogeosci. For..

[3] Jiménez A., Peltzer M.A., Ruseckaite R.A. (2015). Poly(Lactic Acid) Science and Technology: Processing, Properties, Additives and Applications.

[4] Arwinfar F., Hosseinihashemi S.K., Latibari A.J., Lashgari A., Ayrilmis N. (2016). Mechanical Properties and Morphology of Wood Plastic Composites Produced with Thermally Treated Beech Wood. BioResources.

[5] Bledzki A.K., Faruk O., Huque M. (2002). Physico-Mechanical Studies of Wood Fiber Reinforced Composites. Polym. Plast. Technol. Eng..

[6] Nuñez A.J., Sturm P.C., Kenny J.M., Aranguren M.I., Marcovich N.E., Reboredo M.M. (2003). Mechanical Characterization of Polypropylene-Wood Flour Composites. J. Appl. Polym. Sci..

[7] Włodarczyk-Fligier A., Polok-Rubiniec M. (2021). Studies of Resistance of PP/Natural Filler Polymer Composites to Decomposition Caused by Fungi. Materials.

[8] Avérous L., Le Digabel F. (2006). Properties of Biocomposites Based on Lignocellulosic Fillers. Carbohydr. Polym..

[9] Alan K.T.L., Hung A.P.Y. (2017). Natural Fiber-Reinforced Biodegradable and Bioresorbable Polymer Composites.

[10] Bendjaouahdou C., Bensaad S. (2018). Aging Studies of a Polypropylene and Natural Rubber Blend. Int. J. Ind. Chem..

[11] Ashori A. (2008). Wood–Plastic Composites as Promising Green-Composites for Automotive Industries!. Bioresour. Technol..

[12] Thakur V.K., Thakur M.K. (2014). Processing and Characterization of Natural Cellulose Fibers/Thermoset Polymer Composites. Carbohydr. Polym..

[13] Wu J., Yu D., Chan C.M., Kim J., Mai Y.W. (2000). Effect of Fiber Pretreatment Condition on the Interfacial Strength and Mechanical Properties of Wood Fiber/PP Composites. J. Appl. Polym. Sci..

[14] Dányádi L., Móczó J., Pukánszky B. (2010). Effect of Various Surface Modifications of Wood Flour on the Properties of PP/Wood Composites. Compos. Part. A Appl. Sci. Manuf..

[15] Abdulkhani A., Hosseinzadeh J., Ashori A., Dadashi S., Takzare Z. (2014). Preparation and Characterization of Modified Cellulose Nanofibers Reinforced Polylactic Acid Nanocomposite. Polym. Test..

[16] Croitoru C., Varodi A.M., Timar M.C., Roata I.C., Stanciu E.M., Pascu A. (2018). Wood-Plastic Composites Based on HDPE and Ionic Liquid Additives. J. Mater. Sci..

[17] Sahlin K., Forsgren L., Moberg T., Bernin D., Rigdahl M., Westman G. (2018). Surface Treatment of Cellulose Nanocrystals (CNC): Effects on Dispersion Rheology. Cellulose.

[18] Schirp A., Wolcott M.P. (2005). Influence of Fungal Decay and Moisture Absorption on Mechanical Properties of Extruded Wood-Plastic Composites. Wood Fiber Sci..

[19] Feng J., Zhang H., He H., Huang X., Shi Q. (2016). Effects of Fungicides on Mold Resistance and Mechanical Properties of Wood and Bamboo Flour/High-Density Polyethylene Composites. BioResources.

[20] Bari E., Taghiyari H.R., Schmidt O., Ghorbani A., Aghababaei H. (2015). Effects of Nano-Clay on Biological Resistance of Wood-Plastic Composite against Five Wood-Deteriorating Fungi. Maderas. Cienc. Y Tecnol..

[21] Rana A.K., Thakur M.K., Saini A.K., Mokhta S.K., Moradi O., Rydzkowski T., Alsanie W.F., Wang Q., Grammatikos S., Thakur V.K. (2022). Recent Developments in Microbial Degradation of Polypropylene: Integrated Approaches towards a Sustainable Environment. Sci. Total Environ..

[22] Krause K.C., Brischke C., Koddenberg T., Buschalsky A., Militz H., Krause A. (2019). Resistance of Injection Molded Wood-Polypropylene Composites against Basidiomycetes According to En 15534-1: New Insights on the Test Procedure, Structural Alterations, and Impact of Wood Source. Fibers.

[23] Ashori A., Behzad H.M., Tarmian A. (2013). Effects of Chemical Preservative Treatments on Durability of Wood Flour/HDPE Composites. Compos. Part. B Eng..

[24] Feng J., Chen J., Chen M., Su X., Shi Q. (2017). Effects of Biocide Treatments on Durability of Wood and Bamboo/High Density Polyethylene Composites against Algal and Fungal Decay. J. Appl. Polym. Sci..

[25] Pánek M., Reinprecht L., Hulla M. (2014). Ten Essential Oils for Beech Wood Protection—Efficacy Against Wood-Destroying Fungi and Moulds, and Effect on Wood Discoloration. BioResources.

[26] Kwaśniewska-Sip P., Cofta G., Nowak P.B. (2018). Resistance of Fungal Growth on Scots Pine Treated with Caffeine. Int. Biodeterior. Biodegrad..

[27] Teacă C.A., Roşu D., Mustaţă F., Rusu T., Roşu L., Roşca I., Varganici C.D. (2019). Natural Bio-Based Products for Wood Coating and Protection against Degradation: A Review. BioResources.

[28] Akcay C., Birinci E., Birinci C., Kolayli S. (2020). Durability of Wood Treated with Propolis. BioResources.

[29] Fang S., Feng X., Lei Y., Chen Z., Yan L. (2021). Improvement of Wood Decay Resistance with Cinnamaldehyde Chitosan Emulsion. Ind. Crops Prod..

[30] Woźniak M., Kwaśniewska-Sip P., Krueger M., Roszyk E., Ratajczak I. (2020). Chemical, Biological and Mechanical Characterization of Wood Treated with Propolis Extract and Silicon Compounds. Forests.

[31] Casado-Sanz M.M., Silva-Castro I., Ponce-Herrero L., Martín-Ramos P., Martín-Gil J., Acuña-Rello L. (2019). White-Rot Fungi Control on Populus Spp. Wood by Pressure Treatments with Silver Nanoparticles, Chitosan Oligomers and Propolis. Forests.

[32] Silva-Castro I., Diez J.J., Martín-Ramos P., Pinto G., Alves A., Martín-Gil J., Martín-García J. (2018). Application of Bioactive Coatings Based on Chitosan and Propolis for Pinus Spp. Protection against Fusarium Circinatum. Forests.

[33] Ratajczak I., Woźniak M., Kwaśniewska-Sip P., Szentner K., Cofta G., Mazela B. (2018). Chemical Characterization of Wood Treated with a Formulation Based on Propolis, Caffeine and Organosilanes. Eur. J. Wood Wood Prod..

[34] Woźniak M., Mania P., Roszyk E., Ratajczak I. (2021). Bending Strength of Wood Treated with Propolis Extract and Silicon Compounds. Materials.

[35] Woźniak M., Ratajczak I., Lis B., Krystofiak T. (2018). Hydrophobic Properties of Wood Traeted with Propolis-Silane Formulations. Wood Res..

[36] Friedrich D. (2018). Comparative Study on Artificial and Natural Weathering of Wood-Polymer Compounds: A Comprehensive Literature Review. Case Stud. Constr. Mater..

[37] Aydemir D., Alsan M., Can A., Altuntas E., Sivrikaya H. (2019). Accelerated Weathering and Decay Resistance of Heat-Treated Wood Reinforced Polypropylene Composites. Drv. Ind..

[38] La Mantia F.P., Morreale M. (2008). Accelerated Weathering of Polypropylene/Wood Flour Composites. Polym. Degrad. Stab..

[39] Ibach R., Gnatowski M., Sun G., Glaeser J., Leung M., Haight J. (2018). Laboratory and Environmental Decay of Wood–Plastic Composite Boards: Flexural Properties. Wood Mater. Sci. Eng..

[40] Catto A.L., Montagna L.S., Almeida S.H., Silveira R.M.B., Santana R.M.C. (2016). Wood Plastic Composites Weathering: Effects of Compatibilization on Biodegradation in Soil and Fungal Decay. Int. Biodeterior. Biodegrad..

[41] Fabiyi J.S., McDonald A.G., Wolcott M.P., Griffiths P.R. (2008). Wood Plastic Composites Weathering: Visual Appearance and Chemical Changes. Polym. Degrad. Stab..

[42] Gunjal J., Aggarwal P., Chauhan S. (2020). Changes in Colour and Mechanical Properties of Wood Polypropylene Composites on Natural Weathering. Maderas Cienc. Y Tecnol..

[43] Kuka E., Andersons B., Cirule D., Andersone I., Kajaks J., Militz H., Bicke S. (2020). Weathering Properties of Wood-Plastic Composites Based on Heat-Treated Wood and Polypropylene. Compos. Part A Appl. Sci. Manuf..

[44] Naumann A., Stephan I., Noll M. (2012). Material Resistance of Weathered Wood-Plastic Composites against Fungal Decay. Int. Biodeterior. Biodegrad..

[45] Odalanowska M., Woźniak M., Ratajczak I., Zielińska D., Cofta G., Borysiak S. (2021). Propolis and Organosilanes as Innovative Hybrid Modifiers in Wood-Based Polymer Composites. Materials.

[46] Hindeleh A.M., Johnson D.J. (1971). The Resolution of Multipeak Data in Fibre Science. J. Phys. D Appl. Phys..

[47] Rabiej S. (1991). A Comparison of Two X-Ray Diffraction Procedures for Crystallinity Determination. Eur. Polym. J..

[48] Woźniak M., Kwaśniewska-Sip P., Waśkiewicz A., Cofta G., Ratajczak I. (2020). The Possibility of Propolis Extract Application in Wood Protection. Forests.

[49] Kuka E., Cirule D., Kajaks J., Janberga A., Andersone I., Andersons B. (2017). Fungal Degradation of Wood Plastic Composites Made with Thermally Modified Wood Residues. Key Eng. Mater..

[50] Müller M., Gellerich A., Militz H., Krause A. (2013). Resistance of Modified Polyvinyl Chloride/Wood Flour Composites to Basidiomycetes. Eur. J. Wood Wood Prod..

[51] Lis B., Woźniak M., Krystofiak T., Ratajczak I. (2020). Effect of Accelerated Aging on the Color Changes of Wood Treated with Eco-Friendly Formulations Based on Propolis and Sslicon Compounds. BioResources.

[52] Schmidt O. (2006). Wood and Tree Fungi: Biology, Damage, Protection and Use.

[53] Daniel G. (2016). Fungal Degradation of Wood Cell Walls. Secondary Xylem Biology.

[54] Kumar A., Pavla R., Sever S.A., Humar M., Pavlič M., Jan T., Petr H., Zigon J., Petric M. (2016). Influence of Surface Modification of Wood with Octadecyltrichlorosilane on Its Dimensional Stability and Resistance against Coniophora Puteana and Molds. Cellulose.

[55] Pandey K.K., Pitman A.J. (2003). FTIR Studies of the Changes in Wood Chemistry Following Decay by Brown-Rot and White-Rot Fungi. Int. Biodeterior. Biodegrad..

[56] Durmaz S., Özgenç Ö., Boyaci I.H., YIldIz Ü.C., Erişir E. (2016). Examination of the Chemical Changes in Spruce Wood Degraded by Brown-Rot Fungi Using FT-IR and FT-Raman Spectroscopy. Vib. Spectrosc..

[57] Tomak E.D. (2014). Changes in Chemical Composition of Decayed Scots Pine and Beech Wood. Sci. Eng. Compos. Mater..

[58] Pandey K.K., Pitman A.J. (2004). Examination of the Lignin Content in a Softwood and a Hardwood Decayed by a Brown-Rot Fungus with the Acetyl Bromide Method and Fourier Transform Infrared Spectroscopy. J. Polym. Sci. Part A Polym. Chem..

[59] Tomak E.D., Topaloglu E., Gumuskaya E., Yildiz U.C., Ay N. (2013). An FT-IR Study of the Changes in Chemical Composition of Bamboo Degraded by Brown-Rot Fungi. Int. Biodeterior. Biodegrad..

[60] Fackler K., Stevanic J.S., Ters T., Hinterstoisser B., Schwanninger M., Salmén L. (2010). Localisation and Characterisation of Incipient Brown-Rot Decay within Spruce Wood Cell Walls Using FT-IR Imaging Microscopy. Enzym. Microb. Technol..

[61] Can A., Sivrikaya H. (2017). Chemical Characterization of Fungal Deterioration in Populus Alba by FT-IR Chemical Characterization of Fungal Deterioration in Populus Alba by FT-IR. J. Bartin Fac. For..

[62] Mohebby B. (2005). Attenuated Total Reflection Infrared Spectroscopy of White-Rot Decayed Beech Wood. Int. Biodeterior. Biodegrad..

[63] Fackler K., Stevanic J.S., Ters T., Hinterstoisser B., Schwanninger M., Salmén L. (2011). FT-IR Imaging Microscopy to Localise and Characterise Simultaneous and Selective White-Rot Decay within Spruce Wood Cells. Holzforschung.

[64] Akhtari M., Taghiyari H.R., Kokandeh M.G. (2013). Effect of Some Metal Nanoparticles on the Spectroscopy Analysis of Paulownia Wood Exposed to White-Rot Fungus. Eur. J. Wood Wood Prod..

[65] Bari E., Daryaei M.G., Karim M., Bahmani M., Schmidt O., Woodward S., Ghanbary M.A.T., Sistani A. (2019). Decay of Carpinus Betulus Wood by Trametes Versicolor—An Anatomical and Chemical Study. Int. Biodeterior. Biodegrad..

[66] Bari E., Nazarnezhad N., Kazemi S.M., Ghanbary M.A.T., Mohebby B., Schmidt O., Clausen C.A. (2015). Comparison between Degradation Capabilities of the White Rot Fungi Pleurotus Ostreatus and Trametes Versicolor in Beech Wood. Int. Biodeterior. Biodegrad..

[67] Karim M., Daryaei M.G., Torkaman J., Oladi R., Ghanbary M.A.T., Bari E., Yilgor N. (2017). Natural Decomposition of Hornbeam Wood Decayed by the White Rot Fungus Trametes Versicolor. An. Acad. Bras. Cienc..

[68] Popescu C.M., Popescu M.C., Vasile C. (2010). Characterization of Fungal Degraded Lime Wood by FT-IR and 2D IR Correlation Spectroscopy. Microchem. J..

[69] Fackler K., Schwanninger M., Gradinger C., Srebotnik E., Hinterstoisser B., Messner K. (2007). Fungal Decay of Spruce and Beech Wood Assessed by Near-Infrared Spectroscopy in Combination with Uni- and Multivariate Data Analysis. Holzforschung.

[70] Chrissopoulou K., Anastasiadis S.H. (2011). Polyolefin/Layered Silicate Nanocomposites with Functional Compatibilizers. Eur. Polym. J..

[71] Salaita G.N., Ma F.M.S., Parker T.C., Hoflund G.B. (2008). Weathering Properties of Treated Southern Yellow Pine Wood Examined by X-ray Photoelectron Spectroscopy, Scanning Electron Microscopy and Physical Characterization. Appl. Surf. Sci..

[72] Guadagno L., Naddeo C., Vittoria V. (2004). Structural and Morphological Changes during UV Irradiation of the Crystalline Helical Form of Syndiotactic Polypropylene. Macromolecules.

[73] Morancho J.M., Ramis X., Fernández X., Cadenato A., Salla J.M., Vallés A., Contat L., Ribes A. (2006). Calorimetric and Thermogravimetric Studies of UV-Irradiated Polypropylene/Starch-Based Materials Aged in Soil. Polym. Degrad. Stab..

[74] Inagaki T., Siesler H.W., Mitsui K., Tsuchikawa S. (2010). Difference of the Crystal Structure of Cellulose in Wood after Hydrothermal and Aging Degradation: A NIR Spectroscopy and XRD Study. Biomacromolecules.

[75] Lionetto F., Del Sole R., Cannoletta D., Vasapollo G., Maffezzoli A. (2012). Monitoring Wood Degradation during Weathering by Cellulose Crystallinity. Materials.

[76] Couteau C., Pommier M., Paparis E., Coiffard L.J.M. (2008). Photoprotective Activity of Propolis. Nat. Prod. Res..

[77] Karapetsas A., Voulgaridou G.P., Konialis M., Tsochantaridis I., Kynigopoulos S., Lambropoulou M., Stavropoulou M.I., Stathopoulou K., Aligiannis N., Bozidis P. (2019). Propolis Extracts Inhibit UV-Induced Photodamage in Human Experimental in Vitro Skin Models. Antioxidants.

[78] Galeotti F., Maccari F., Fachini A., Volpi N. (2018). Chemical Composition and Antioxidant Activity of Propolis Prepared in Different Forms and in Different Solvents Useful for Finished Products. Foods.

[79] Afiqah N., Zafiah M.R.A., Rus M., Zulhafiz M., Syah A.S., Hazwanee F., Aida S., Afiqah S.N. (2018). Mechanical Properties of Wood Polymer Composites (WPCs) After Prolonged Ultra Violet (UV) Irradiation Exposure. Int. J. Eng. Technol..

[80] Seldén R., Nyström B., Långström R. (2004). UV Aging of Poly(Propylene)/Wood-Fiber Composites. Polym. Compos..

[81] Curling S.F., Clausen C.A., Jerrold W.E. (2002). Relationships Between and Chemical Composition of Wood During Incipient Brown-Rot Decay. For. Prod. J..

