# Supplementary figures and images for: Bioactive Propolis-Silane System as Antifungal Agent in Lignocellulosic-Polymer Composites
Source: Materials (Basel). 2022 May 10;15(10):3435. doi: 10.3390/ma15103435 (PMC9145847; doi:10.3390/ma15103435)

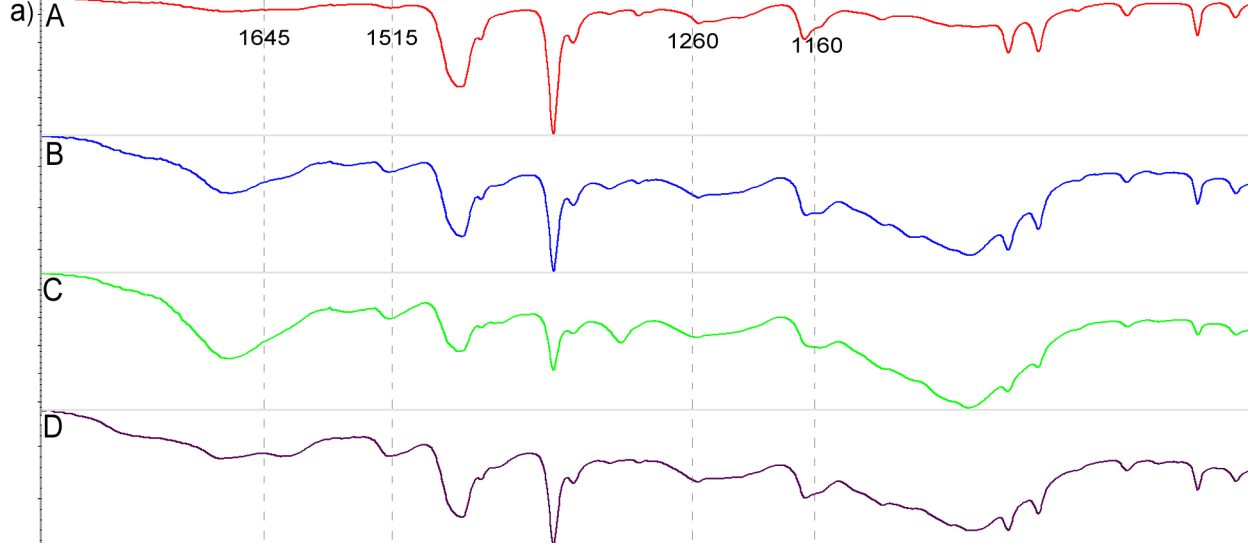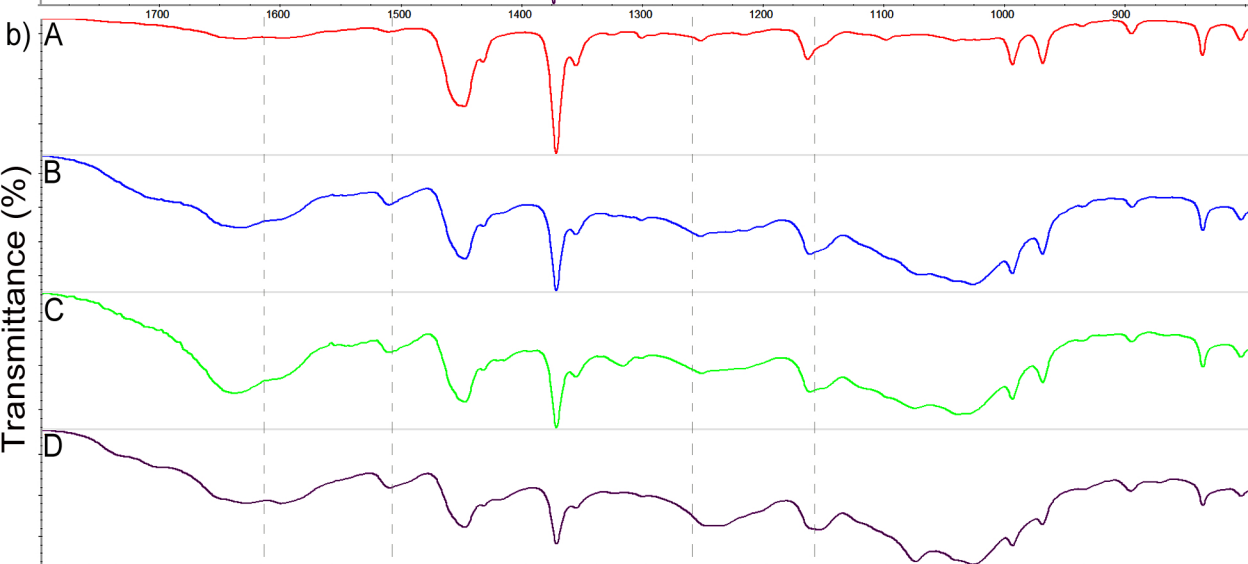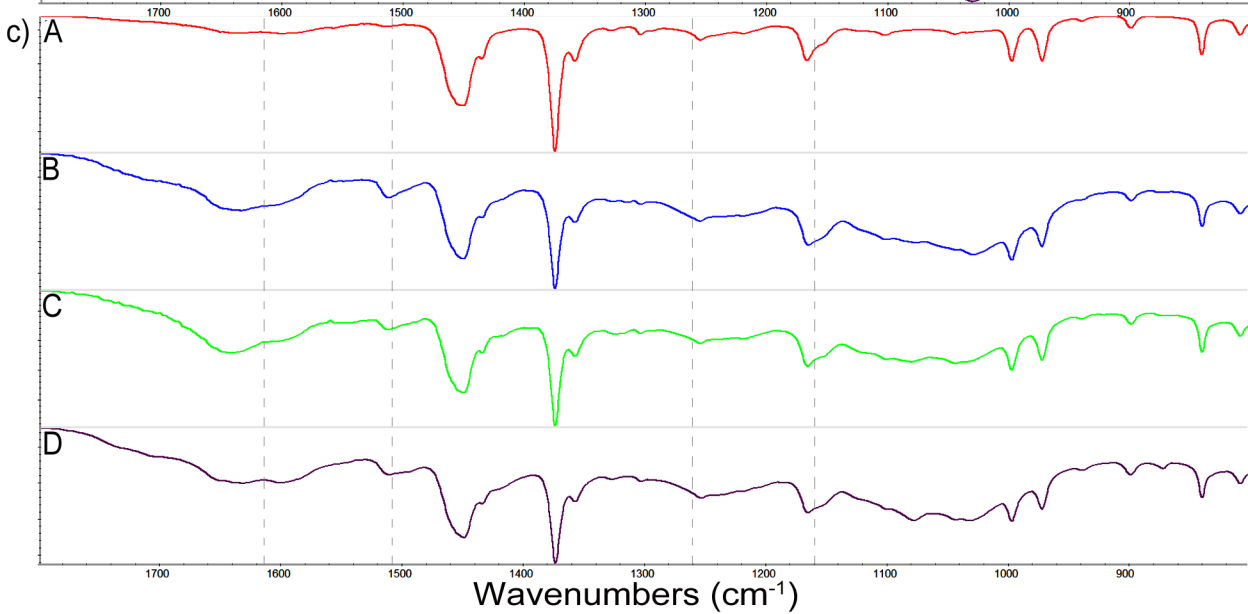

Supplement: Supplementary file 1 [file materials-15-03435-s001.zip › Figure S1.pdf]

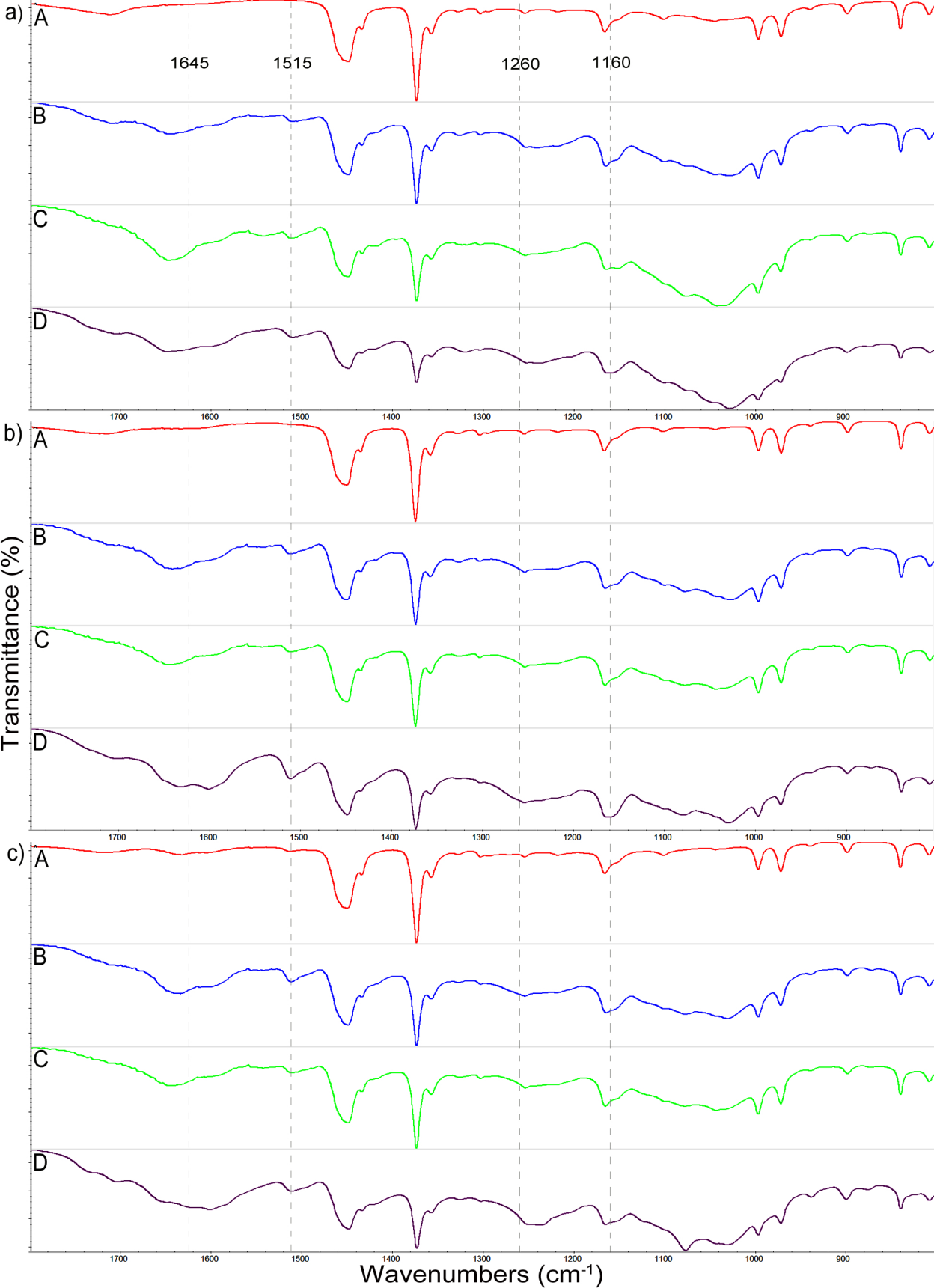

Supplement: Supplementary file 1 [file materials-15-03435-s001.zip › Figure S2.pdf]
